# HIV infection and cardiovascular risk in black South Africans

**Published:** 2011-06

**Authors:** CMT Fourie, JM Van Rooyen, AE Schutte

**Affiliations:** Hypertension in Africa Research Team (HART) North-West University, Potchefstroom, South Africa; Hypertension in Africa Research Team (HART) North-West University, Potchefstroom, South Africa; Hypertension in Africa Research Team (HART) North-West University, Potchefstroom, South Africa

The overall growth of the global AIDS epidemic appears to have stabilised and the number of new infections is declining.^1^ This and the significant reduction in mortality could be attributed to the effectiveness of antiretroviral therapy (ART). Human immunodeficiency virus (HIV) infection, although still fatal, has become a chronic and manageable disease. The therapy has increased the life expectancy of HIV-infected individuals and therefore more people are living with HIV. The region affected the most by HIV remains sub-Saharan Africa, and South Africa continues to be the country housing the largest population of people (an estimated 5.6 million people in 2009) living with HIV worldwide.^1^

Besides some very uncomfortable side effects due to both HIV infection and the therapy, another more serious side effect has emerged, namely an increased risk for cardiovascular disease (CVD).^2^,^3^ HIV infection paradoxically affects cardiovascular risk factors and circulatory disease within populations and individuals. Researchers have associated HIV infection and especially the use of ART with an increase in insulin resistance, dyslipidaemia,^4^ lipodystrophy^5^,^6^ endothelial dysfunction,^7^ accelerated atherosclerosis^8^ and coagulation disorders.^9^

In the past 15 years South Africa has experienced a rise in noncommunicable diseases, such as cardiovascular disease, which is predicted to increase in the next decades.^10^ This rise in incidence of non-communicable diseases is masked by the overwhelming presence of communicable diseases such as HIV and tuberculosis.^11^ Therefore, cardiovascular complications in the HIV-infected population could become a serious health problem in South Africa by increasing the burden of non-communicable diseases once patients are receiving ART for longer periods.^11^ Recent research has shown that atherosclerotic disease, historically not common in black Africans, is increasing in South Africa.^12^

The South African HIV-infected population has had access to free antiretroviral treatment since 2004 and the influence of ART on the cardiovascular system in this population is not yet established. The South African National AIDS Council updated the HIV treatment guidelines and adopted some of the recent recommendations made by the World Health Organisation (WHO), which will lead to more people receiving treatment.^13^ This expansion of antiretroviral therapy and the effect thereof on the burden of non-communicable diseases (such as cardiovascular disease) in South Africa is yet to be determined.

The predominant virus responsible for the infections in South Africa is HIV-1, group M (major), subtype C,^14^,^15^ which accounts for 55 to 60% of all HIV-1 infections worldwide,^16^ and differs as much as 30% in its genome from HIV-1 subtype B, responsible for most infections in North America, Europe and Australia.^14^,^16^,^17^ The clinical consequences of these subtype variations remain unclear.^18^

Although the risk for the development of cardiovascular disease has been described in many different HIV-1-infected populations, data on the risk facing the South African HIV-infected population is scarce, as most of the research is done on Caucasians infected with HIV-1 subtype B. Therefore, the risk of cardiovascular disease in HIV-infected South Africans and how it is affected by the roll-out programme of ART remains largely unknown. The various effects of the virus itself – including its cardiovascular effects – are also important, since many HIV-infected South Africans are still without therapy and/or unaware of their infection status. Various factors seem to contribute to the latter, such as the lack of knowledge, poverty, stigma, scepticism and lack of interest.^19^

The Hypertension in Africa Research Team (HART) therefore individually matched 300 newly identified HIV-infected Africans from the South African Prospective Urban and Rural Epidemiology (PURE) study with 300 uninfected controls. They were matched according to age, gender, body mass index and locality (urban/rural). The larger PURE study is an epidemiological study that will address questions regarding the cause and development of cardiovascular risk factors and disease within populations, including South Africa.^20^ A minimum follow up of 10 years is planned. The South African leg of the study was performed in the North West Province where a total of 2 010 participants (1 004 urban and 1 006 rural) were randomly recruited from a rural and urban setting and screened during the baseline phase in 2005. A follow up on the PURE South Africa study was done in 2010. The newly identified HIV-infected participants and their controls of the baseline (2005) study were also followed up in 2008.

In a cross-sectional analysis on the baseline data we aimed to evaluate if HIV-1 infection itself is associated with dyslipidaemia, inflammation and the occurrence of the metabolic syndrome in newly identified HIV-1-infected black South Africans who had never received antiretroviral therapy. We concluded that HIV-1 is associated with dyslipidaemia and an inflammatory state in newly identified HIV-infected, never-treated African individuals and that it may increase their risk for cardiovascular disease. The study showed that HIV-1, most likely subtype C, seems to influence the components of the metabolic syndrome in South Africans in the same way as HIV-1 subtype B does in Caucasians. It also showed that the virus does not increase the prevalence of the metabolic syndrome in these never-treated, HIV-infected South Africans.^21^

In this edition of the *Cardiovascular Journal of Africa*, a study is published in which we assessed cross-sectionally whether these newly identified, never-treated, HIV-1-infected Africans showed signs of inflammatory injury of the endothelium. This could lead to endothelial dysfunction, accelerated atherosclerosis and increased coagulation, which could result in thrombosis. Our findings suggest inflammatory injury of the endothelium, which was probably worsened by the attenuation of the protective effect of high-density lipoprotein cholesterol (HDL-C). The high levels of protective HDL-C in black South Africans is thought to be the reason for the fairly low prevalence of ischaemic heart disease in the general population.^22^ Although there was no indication of a prothrombotic state which could result in atherosclerotic disease, there was an indication of accelerated vascular aging and probable early atherosclerosis in the older HIV-infected South Africans. The latter indicates a decrease in vascular function of the never-treated, HIV-1-infected older population.^23^

After being identified as HIV infected, the participants were referred to their nearest hospital or clinic for follow up on the diagnosis of HIV infection and commencement of treatment if needed. Some of the participants were eligible, by CD_4_ cell count, for enrollment in the ART roll-out programme but chose not to initiate treatment. During the 2008 follow up, our results showed lower systolic blood pressure and dyslipidaemia in the never-treated, HIV-1-infected South Africans compared to the control participants. In the treated HIV-infected participants, we observed an increase in systolic blood pressure, but no hypertension, and an improvement in lipid profile.

Although the antiretroviral treatment stabilised the lipid profile, an increase in lipodystrophy was seen in the treated group, which may influence the development of future cardiovascular disease. Indeed, changes in body composition were one of the most prominent results of this study. These changes in the treated group confirm the possible development of lipodystrophy, expected after the introduction of antiretroviral treatment.^5^,^6^ Stavudine, the nucleoside reverse transcriptase inhibitor (NRTI) of the first-line therapy of the roll-out programme is incriminated in the development of lipodystrophy.^24^ New guidelines on the WHO’s first-line therapy, which came into effect on 1 April 2010 in South Africa, phase out the use of stavudine in favour of tenofovir.^13^ Whether this could have an effect on the development of lipodystrophy in the South African population remains to be seen.

The novel biomarker, soluble urokinase plasminogen activator receptor (suPAR) is a stable plasma protein^25^ and is associated with inflammation and progression of disease in HIV-1 infection.^26^ It was suggested that suPAR may be a marker linking inflammatory and metabolic characteristics (lipid and glucose metabolism, as well as fat redistribution) of HIV-infected patients on ART.^25^ We therefore hypothesised that the HIV-1-infected black South Africans would have significantly higher suPAR levels than their uninfected controls. While the latter was confirmed by our results, the treated HIV-1-infected Africans unexpectedly showed a significantly greater increase in blood suPAR levels than never-treated infected or uninfected Africans after a three-year period. Furthermore, this study indicates an association of suPAR with the development of lipodystrophy in HIV-1-infected black South Africans on the WHO’s recommended first-line antiretroviral therapy.^27^

In summary, our results clearly indicate a detrimental health profile in the HIV-infected black population of South Africa (whether receiving treatment or not). Therefore it remains of the utmost importance to gain knowledge about the influence of HIV infection and the treatment thereof on the cardiovascular system of the South African population.

## References

[1] Aids epidemic update. Regional summary 2010. http://www.unaids.org/documents/20101123_GlobalReport_em.pdf..

[2] De Gaetano DK, Rabagliati R, Iacoviello L (2004). HIV infection, HAART, and endothelial adhesion molecules: current perspectives.. Lancet Infect Dis.

[3] Fisher SD, Miller TL, Lipshultz SE (2006). Impact of HIV and highly active antiretroviral therapy on leukocyte adhesion molecules, arterial inflammation, dyslipidemia, and atherosclerosis.. Atherosclerosis.

[4] Riddler SA, Smit E, Cole SR (2003). Impact of HIV infection and HAART on serum lipids in men.. J Am Med Asssoc.

[5] Carr A, Cooper DA (1998). Images in clinical medicine. Lipodystrophy associated with an HIV-protease inhibitor.. N Engl J Med.

[6] Grinspoon SK (2005). Metabolic syndrome and cardiovascular disease in patients with human immunodeficiency virus.. Am J Med.

[7] Constans J, Conri C (2006). Circulating markers of endothelial function in cardiovascular disease.. Clin Chim Acta.

[8] Hsue PY, Lo JC, Franklin A (2004). Progression of atherosclerosis as assessed by carotid intima-media thickness in patients with HIV infection.. Circulation.

[9] Glazier JJ, Spears JR, Murphy MC (2006). Interventional approach to recurrent myocardial infarction in HIV-1 infection.. J Interv Cardiol.

[10] Abegunde DO, Mathers CD, Adam T (2007). The burden and costs of chronic diseases in low-income and middle-income countries.. Lancet.

[11] Mayosi BM, Flisher AJ, Lalloo UG (2009). The burden of non-communicable diseases in South Africa.. Lancet.

[12] Sliwa K, Wilkinson D, Hansen C (2008). Spectrum of heart disease and risk factors in a black urban population in South Africa (the Heart of Soweto Study): a cohort study.. Lancet.

[13] The South African antiretroviral treatment guidelines. 2010.. http://www.epiresult.com/epidemiology-news/23/updated-art-treatment-guidelineswho-and-south-africa.html..

[14] Peeters M (200). The genetic variability of HIV-1 and its implications.. Transfus Clin Biol.

[15] Jacobs GB, de Beer C, Fincham JE (2006). Serotyping and genotyping of HIV-1 infection in residents of Khayelitsha, Cape Town, South Africa.. J Med Virol.

[16] Freire E (2006). Overcoming HIV-1 resistance to protease inhibitors.. Drug Discovery.

[17] Gaschen B, Taylor J, Yusim K (200). Diversity considerations in HIV-1 vaccine selection.. Science.

[18] Simon V, Ho DD, Abdool KQ (2006). HIV/AIDS epidemiology, pathogenesis, prevention, and treatment.. Lancet.

[19] Kruger A, Greeff M, Watson MJ, Fourie CMT (2009). Health care seeking behaviour of newly diagnosed HIV infected people from rural and urban communities in the North West province of South Africa.. Afr J Nurs.

[20] Teo K, Chow CK, Vaz M (2009). The Prospective Urban Rural Epidemiology (PURE) study: Examining the impact of societal influences on chronic noncommunicable diseases in low-, middle-, and high-income countries.. Am Heart J.

[21] Fourie CM, van Rooyen JM, Kruger A, Schutte AE (2010). Lipid abnormalities in a never -treated HIV-1 subtype C-infected African population.. Lipids.

[22] Seedat YK, Mayet FGH, Latiff GH (1993). Study of risk factors leading to coronary heart disease in urban Zulus.. J Hum Hypertens.

[23] Fourie CM, van Rooyen JM, Pieters M (2011). Is HIV-1 associated with endothelial dysfunction in a population of African ancestry in South Africa?. Cardiovasc J Afr.

[24] Domingo P, Cabeza MC, Pruvost A (2010). Relationship between HIV/highly active antiretroviral therapy (HAART)-associated lipodystrophy syndrome and stavudine-triphosphate intracellular levels in patients with stavudine-based antiretroviral regimens.. Clin Infect Dis.

[25] Andersen O, Eugen-Olsen J, Kofoed K (2008). suPAR associates to glucose metabolic aberration during glucose stimulation in HIV-infected patients on HAART.. J Infect.

[26] Sidenius N, Sier CF, Ullum H, Pedersen BK, Lepri AC, Blasi F (2000). Serum level of soluble urokinase-type plasminogen activator receptor is a strong and independent predictor of survival in human immunodeficiency virus infection.. Blood.

[27] Fourie CM, Van Rooyen JM, Kruger A (2011). Soluble urokinase plasminogen activator receptor (suPAR) is associated with metabolic changes in HIV-1-infected Africans: A prospective study.. Inflammation.

